# Supplementary material for: Patterns of Intron Gain and Loss in Fungi
Source: PLoS Biol. 2004 Nov 30;2(12):e422. doi: 10.1371/journal.pbio.0020422 (PMC532390; doi:10.1371/journal.pbio.0020422)
Supplement: Table S1 — Also available at http://genes.mit.edu/NielsenEtAl/. (4.3 MB ZIP). [file pbio.0020422.st001.zip › NielsenEtAl/html/154.html]

AN5865.1.NCU05289.1.MG07136.1.FG06165.1


```
 CLUSTAL W (1.82) Multiple Sequence Alignments - Introns Inserted


Sequence 1: AN5865.1	656 aa
Sequence 2: FG06165.1	658 aa
Sequence 3: MG07136.1	659 aa
Sequence 4: NCU05289.1	666 aa
Alignment Length: 669 aa
Number Identitical Residues: 373 aa
Alignment Score (without introns) 17848


MG07136.1 	MKTTWKD~IPPVPT~SQEFLDIVLS~RTQRRLPTQI~RAGFKI~SRIRG~FYTRKVKFTQ
NCU05289.1	--MTWKD~IAPVPT2AQEFIDIILS2RTQRRLPTQI1RAGFKI1SRIRA~FYTRKVKFTQ
FG06165.1 	MKTTWKD~IPPVPT~QQEFLDIVLS~RTQRKLPTQI~RAGFKI~SRIRA1FYTRKVKFTQ
AN5865.1  	MKTTWKD0IPPVPT~SQEFLDIVLS~RTQRQLPTQI~RAGFKI~SRIRG1FYTRKVKYTQ
          	 . **** *.****  ***:**:** ****:***** ****** ****. *******:**

MG07136.1 	ET2FSEKFEAILSSFPRILDLHPFH~KDLLNTLYD~ADHFKVALGQVSTAKHLIETISRD
NCU05289.1	ET~CSEKFGAIVSSFPILADQHPFH~RDLMNILYD1ADHFKVALGQVSTAKHLIETISRD
FG06165.1 	ET~FSEKFGAILESFPRLQDIHPFH~KDLLNTLYD~ADHFRIALGQMSTAKHLIETISRD
AN5865.1  	ET~FGEKFQAILDGFPRLQDIHPFH1KDLMNTLYD~ADHFRIALGQVSTAKHLIETVSRD
          	**  .*** **:..** : * **** :**:* *** ****::****:*********:***

MG07136.1 	YVRLLKYGQSLYQCRQLKVAALGRMATICKKLKDSLVYLDQVRQH2LGRLPSIDPTTRTL
NCU05289.1	YVRLLKYSQSLYQCKQLKRAALGRMATLIKRLKDPLAYLDQVRQH~LARLPDINPTTRTL
FG06165.1 	YVRLLKYGQSLFQCKQLKRAALGRMATLIKRLKDPLMYLDQVRQH~LGRLPSIDPNTRTL
AN5865.1  	YVRLIKYAQSLFQCKQLKRAALGRMATICRRLKDPLVYLEQVRQH~LGRLPSIDPNTRTL
          	****:**.***:**:*** ********: ::***.* **:***** *.***.*:*.****

MG07136.1 	LIAGFPNVGKSSFLKSVTRADVDVQPYAFT1TKSLFVGHLDYKYLKFQVIDTPGILDHPL
NCU05289.1	LVAGFPNVGKSSFVRSVTRADTPVEPYAFT~TKSLFVGHLDYKYLRYQVIDTPGILDHPL
FG06165.1 	LICGYPNVGKSSFLKSITRADVDVQPYAFT~TKSLFVGHFDYKYLRFQAIDTPGILDHPL
AN5865.1  	LICGYPNVGKSSFLRSITRADVDVQPYAFT~TKSLFVGHFDYKYLRFQAIDTPGILDHPL
          	*:.*:********::*:****. *:***** ********:*****::*.***********

MG07136.1 	DEM0NTIEMQSITAIAHLRSVILYFMDLSEQCGYTVQAQMQLFRSIKPLFANKIVFIVVN
NCU05289.1	EEM~NTIEMQSVTALAHLRAAVMFFIDISEQCGYSLKAQCNLFRSIKPLFENKMVYVVLN
FG06165.1 	EEM~NTIEMQSITAIAHLRSAIMYFMDLSEQCGYTVSAQIALFKSIKPLFSNKLVFVVIN
AN5865.1  	EEM~NTIEMQSITAIAHLRSAVMYFMDLSEQCGYSVADQIKLFHSIRPLFANKIVFVVVN
          	:** *******:**:****:.:::*:*:******::  *  **:**:*** **:*::*:*

MG07136.1 	KIDVMKPEDLDAKLQEELQALLKSDGVEMLQLSCNTQEGVQAVKNAACEKLIAERVAQKL
NCU05289.1	KMDIKTVEDLDPESQADLMDLTKSGNIQLLRASCATQDGVQEVKNTVCEALLVERVNQKY
FG06165.1 	KIDVTRPEDLEPSVQEELQGILKSGEVELLQLSCNTMEGVQEVKNAACERLIAERVNQKL
AN5865.1  	KIDVRRPEDLEPELQEQMQSMLKTGDVELLQLSCTTTEGVTNVKNAACDKLLAERVAQKL
          	*:*:   ***:.. * ::  : *:. :::*: ** * :**  ***:.*: *:.*** ** 

MG07136.1 	KAGTTSSGAAGGRLADVMARIHVAQPMGGSQLETFVPEGIKQLKKYDKADPERRLLARDI
NCU05289.1	KAGTSSNGTMGSRLTEVMSRIHVAQPADGVLRETFIPEAVKGLKKYDKADPERKLLARDI
FG06165.1 	KAGTNTSGAIGGRLADVMSRIHVAQPMGGQTLETFIPEGIKDRKKYDKEDPERRRTARDE
AN5865.1  	KSGTNSTGTPGGRLGDVLARIHVAQPMGGAQLETFIPDAVKNLKKYDKNDPNRKRLERDI
          	*:**.:.*: *.** :*::******* .*   ***:*:.:*  ***** **:*:   ** 

MG07136.1 	EEMNGGAGVFNVDLREKWILDNPEWKYDKIPE~VFDGKNVYDYVDPEIEAKLAALEAEEE
NCU05289.1	EEQNGGAGVFNVDLRENWILANPEWKYDKIPE0IVDGKNVYDFIDPDIEAKLAALEEEEE
FG06165.1 	QDENGGAGVFNVDMKADYMLANPEWKYDKIPE~IYDGKNVYDFIDPDIEAKLQALEEEEE
AN5865.1  	EEENGGAGVYNVDLKKSYTLADDEWKHDKIPE~VWNGKNIYDYVDPDIEAKLAALEEEEE
          	:: ******:***:: .: * : ***:***** : :***:**::**:***** *** ***

MG07136.1 	KLEAEGYYNSDEEIVDDEEEEVMAKAELIREKQSMIRNEAKMKKSLKNRAIIPRKATKKS
NCU05289.1	RLEKEGFYKSDSDLGDESEEEILQKAEYIREKHKLIRNEAKMKKSLKNRAIIPRKMQKKS
FG06165.1 	KLEAEGFYESDEDDEDDEEAEVLRKAELIREKQQLIRNEARMKKRLKNQAIIPRKMMKKP
AN5865.1  	KLEADGYYDSDESVEDAEDADVRMKADLIREKRTLMRNEAKLRKSLKNRAAIPRSAKAKK
          	:** :*:*.**..  * .: ::  **: ****: ::****:::* ***:* ***.   * 

MG07136.1 	LVQMEDALDQLGVDTTDITARARAQSRP--RGRSLTRSRMGTEDIDSMDIDGRAKTPRER
NCU05289.1	FAQLEDHIDQLGVDTEEINLRGRAQVREPTRGRSLARSRNATEDPDAMEVDT-PKSAAER
FG06165.1 	LSEFEDALDQLGVDTTDIADRARERSRG--RAR--SRSRMGTEDPDSMDVDGHTSTPRER
AN5865.1  	LSQMEQGLDAAGYDPEAAVSRVRSQSQV--RGR--TTTRSEVEDGDAMDID--PSDPRQA
          	: ::*: :*  * *.     * * : :   *.*  : :*  .** *:*::*  .. . : 

MG07136.1 	LRSKS-RARSQP-ATDRRNDGVA------DESVRTAAERQAKLSQRKMNRMARQGEADRH
NCU05289.1	LRSQSRPAQRGQGATNRRDDGVTGGVGMDGETARSKAERVAKLGQRKMNRMARAGEADRH
FG06165.1 	MRSISRPASRAP-TASRRDDGVP------DDVLRSKADRIAKLNQKRMNRMARQGEGDRH
AN5865.1  	IARAKGRARSQA-ATNRLQDGVT------DETARSKAERLAKLGQKKMNRMARQGEADRH
          	:   .  *     ::.* :***.      .:  *: *:* ***.*::****** **.***

MG07136.1 	ATASIAKHL0FSGKRGMGKTQRR
NCU05289.1	IGATMPKHL0FSGKRTVGKTQRR
FG06165.1 	TTASITKHL0VVGKRGMGKTNRR
AN5865.1  	QTVSLTKHL0VAGKRGLGKTQRR
          	  .::.*** . *** :***:**
```
